# Do people who choose their treatment in a large randomised trial with parallel preference groups differ at baseline from those who agree to random treatment allocation? Results from the protect study

**DOI:** 10.1186/1745-6215-16-S2-P109

**Published:** 2015-11-16

**Authors:** Nicola Mills, Hanan Khazragui, Chris Metcalfe, Athene Lane, Michael Davis, Grace Young, David Neal, Freddie Hamdy, Jenny Donovan

**Affiliations:** 1 University of Bristol, Bristol, UK; 2 University of Cambridge, Cambridge, UK; 3 University of Oxford, Oxford, UK

## Background

Many patients decline participation in randomised trials due to treatment preferences. It is not known whether it is possible to combine evidence from those choosing treatment and those randomised in RCTs with parallel non-randomised preference groups without compromising external validity. This study explored whether people who chose their treatment within an RCT in which recruiters were trained to explore treatment preferences were different at baseline from those who agreed to randomisation.

## Methods

The study population consisted of men aged 50-69 years with localised prostate cancer detected as part of the ProtecT study. Patients who consented to randomisation and those who selected a treatment instead were assessed for socio-demographic, clinical and health measures at baseline. Logistic regression was used to compare characteristics between men who consented to randomisation and those who chose a treatment.

## Results

Few clear differences between those in the randomised and non-randomised groups were found except those who chose their treatment were more affluent, based on occupation and postcode, and less anxious than those randomised. No differences in clinical measures were identified.

## Conclusions

This study suggests that patient treatment preferences may not seriously compromise the external validity of an RCT. We can be confident from previous research on this population that we were comparing those who held clear and informed preferences with those who held no or only very weak preferences. Training recruiters to optimise informed consent and recruitment to ensure as many patients as possible have the option of randomisation may make preferences less problematic in trials.

